# Beyond HAT Adaptor: TRRAP Liaisons with Sp1-Mediated Transcription

**DOI:** 10.3390/ijms222212445

**Published:** 2021-11-18

**Authors:** Bo-Kun Yin, Zhao-Qi Wang

**Affiliations:** 1 Leibniz Institute on Aging-Fritz Lipmann Institute (FLI), 07745 Jena, Germany; Bokun.Yin@leibniz-fli.de; 2 Faculty of Biological Sciences, Friedrich Schiller University Jena, 07743 Jena, Germany

**Keywords:** TRRAP, HAT, Sp1, transcription, neuro-development, neuodegeneration

## Abstract

The members of the phosphatidylinositol 3-kinase-related kinase (PIKK) family play vital roles in multiple biological processes, including DNA damage response, metabolism, cell growth, mRNA decay, and transcription. TRRAP, as the only member lacking the enzymatic activity in this family, is an adaptor protein for several histone acetyltransferase (HAT) complexes and a scaffold protein for multiple transcription factors. TRRAP has been demonstrated to regulate various cellular functions in cell cycle progression, cell stemness maintenance and differentiation, as well as neural homeostasis. TRRAP is known to be an important orchestrator of many molecular machineries in gene transcription by modulating the activity of some key transcription factors, including E2F1, c-Myc, p53, and recently, Sp1. This review summarizes the biological and biochemical studies on the action mode of TRRAP together with the transcription factors, focusing on how TRRAP-HAT mediates the transactivation of Sp1-governing biological processes, including neurodegeneration.

## 1. The PIKK Family and TRRAP

**Figure 1:**
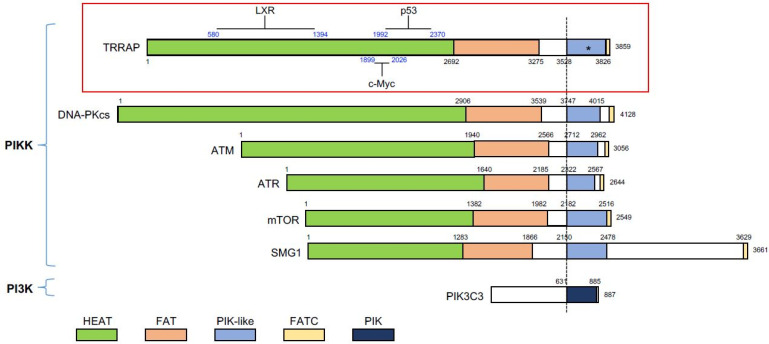
Protein domains of the phosphatidylinositol 3-kinase-related kinase (PIKK) family members. All members contain the PIK-like domain that confers kinase activity, except TRRAP that lacks the key motifs (marked with asterisk). The PIK-like domain is flanked by the FAT domain and the FATC domain. PIK3C3, a member of phosphoinositide 3-kinases (PI3K), serves as a comparison. The known regions (blue numbers) in TRRAP, which interact with p53, c-Myc, and LXR (liver X receptor), are shown. Many of the proteins have been reported to associate with TRRAP; however, if there are no exact regions mapped, these proteins are not shown. The black numbers indicate the amino acid sequence of TRRAP. All of the proteins were aligned with the dashed line, which indicates the N-terminal border of the PIK-like/PIK domain. PIKK, phosphatidylinositol 3-kinase-related kinase; PI3K, phosphoinositide 3-kinases; PIK3C3, phosphatidylinositol 3-kinase catalytic subunit type 3.

Phosphatidylinositol 3-kinase-related kinases (PIKK) are a protein family consisting of six serine/threonine protein kinases that transfer signals to a variety of protein substrates to control multiformities of biological processes. PIKK members share a structural commonality and identity in protein domains. Each PIKK member contains the HEAT repeat, FAT domain, and FATC motif (Figure 1). Although the catalytic domain shares homology between the PIKK and the PI3K family, the PIKK catalytic motif varies from PI3K in the structure of the ATP-binding motif, VAIK, and the divalent cation-binding motif, DFG. Functionally, PIKKs are not involved in lipid phosphorylation, as is the major task of PI3K [1] (Figure 1). Despite similar biochemical function of the PIKK members, the biological functions of these members are very diverse. DNA-PKcs, ATM, and ATR are considered to be important regulators in DNA damage response (DDR) and DNA repair [2]. mTOR is a nutrient-responding kinase, which regulates pathways in metabolism and cell growth [3]. SMG1 is involved in mRNA decay, a process of preventing an accumulation of toxic RNA species due to a premature stop codon or the presence of aberrant 3′-UTR [4]. TRRAP, a short term for transformation/transcription domain-associated protein, is the only pseudokinase in this family, which was initially identified as a protein involved in oncogenic transformation and plays an essential role in gene transcription [5]. Generally, the kinase domain without its catalytic activity, if conserved throughout evolution, is considered the fundamental importance of the non-catalytic function of the protein [6].

TRRAP is a huge protein consisting of 3859 amino acids [1] of 434 kD molecular weight. Initially, TRRAP was identified as an essential co-activator of c-Myc and E2F1 [7]. Later studies showed that all orthologs and paralogs of TRRAP (yeast ortholog Tra1) lack the enzymatic activity. Three motifs for the PIKK kinase activity are missing in the PI3K-like kinase domain in TRRAP: the ATP-binding motif VAIK, the catalytic motif HRD, and the divalent cation-binding motif (DFG) [7,8]. Phylogenetic analysis claims that TRRAP/Tra1 is highly conserved through the eukaryotic clades and considered it as the ancestral member of the PIKK family [1].

## 2. TRRAP and HAT in Transcription

### 2.1. TRRAP as an Adaptor Protein of HAT

Lysine acetylation on histone tails leads to the relaxation of chromatin structure, granting accessibility of transcription factors (TFs) and transcription machinery to the chromatin. The chromatin dynamics are influenced through various post-translational modifications, including phosphorylation, methylation, acetylation, SUMOylation [9], ADP-ribosylation, UFMylation, and serotonylation. Acetylation of the lysine residue on histones is mainly carried out by the activity of histone acetyltransferase (HAT or KAT, thereafter refer to HAT) and is a major epigenetic modulation of chromatin remodeling and gene expression. In a reversible way, the acetylated protein can be deacetylated through histone deacetylases (HDAC or KDAC, thereafter refer to HDAC). The different subunits of the HAT complexes include acetyltransferases, HAT adaptors, interactors of transcription machineries, and also other post-translational modification enzymes, e.g., for histone deubiquitination [10]. The biological function of the subunits within each HAT complex still remains largely unknown, but it is believed that adaptors may dictate the function of individual HAT complexes.

A plethora of studies identified orthologs and paralogs of TRRAP as a shared component of several HAT complexes in eukaryotes, from yeast to humans. TRRAP is in association with two major HAT families: the general control nonderepressible-related (GCN5) acetyltransferase (GNAT) HAT family (including Gcn5 and PCAF) and the MOZ, Ybf2/Sas3, Sas2, Tip60-related (MYST) HAT family (including Tip60) [11]. TRRAP is a subunit of two megamolecular complexes: the Spt-Ada-Gcn5 acetyltransferase complex (named SAGA both in yeasts and mammals) and the nucleosome acetyltransferase of H4 complex (named NuA4 in yeasts and TIP60 in mammals) [1]. Although TRRAP-containing HAT complexes consist of different subunits and acetylate different substrates, TRRAP is likely to be the only shared component among different HAT complexes, for instance within SAGA and TIP60 complexes [12].

### 2.2. The Role of TRRAP in Transcription

In mammals, TRRAP has been shown to interact with multiple TFs, including E2F1, c-Myc, p53, LXR, and *β*-catenin, leading to the transcription activation of their target genes [7,13,14,15,16] (Figure 1). Histone acetylation has later been demonstrated to form docking sites for TFs [17]. Although there is no direct evidence showing that TRRAP interacts with chromatin in a direct manner, a new study on the 3D structure of the yeast TRRAP ortholog, Tra1, suggested that TRRAP owns a DNA binding domain [18]. Although the 3D structure of murine TRRAP has also been mapped (UniProt ID: Q80YV3), whether the DNA binding region affects the interaction between TRRAP-chromatin and their recruitment of TFs, is currently unknown.

As TRRAP is an adaptor protein in HAT complexes, many studies have focused on the TRRAP-related HAT activity and transcription activation. Early studies showed that TRRAP-containing HAT complexes are recruited to an activator-interacting nucleosome to initiate transcription [19,20]. Mechanistically, TRRAP recruits HAT and TFs to chromatin, leading to the hyperacetylation of the histone and activates the transcription of the target genes. Depending on the model of the studies, the target genes can be responsible for different cellular processes, depending on the cell types (Table 1).

TRRAP is an essential co-activator of c-Myc and E2F and the knockdown of TRRAP inhibits the c-Myc- and E1A-mediated oncogenic transformation [7]. In cooperation with p53, TRRAP activates MDM2 transcription through the recruitment of p53 and by increasing histone acetylation on the MDM2 promoter [13]. *β*-catenin associates with TRRAP and mixed-lineage-leukemia (MLL1/MLL2) SET1-type chromatin-modifying complexes, which lead to H3K4 trimethylation on the Wnt target gene c-Myc and the transactivation of *β*-catenin [15]. TRRAP interacts with and co-activates LXR*β*, an activator controlling lipid metabolism, which then induces the expression of LXR*α* targets: ATP-binding cassette transporters A1 and G1 (ABCA1 and ABCG1), stearoyl-CoA desaturase (SCD), and high-density lipoprotein binding protein (HBP) [16]. TRRAP is a component of the PGC-1*β* transcriptional complex to regulate the APOC3 expression and thereby, the lipoprotein metabolism [21]. The co-activator of histone transcription, NPAT, recruits the TRRAP-Tip60 complex to the promoter of H2B and H4 and activates the transcription of histone genes during the G1/S-phase transition [24]. As it is upstream of the transcriptional coregulator Multicilin, TRRAP regulates the expression of MCIDAS, CCNO, and Myb, which are involved in multiciliated cell differentiation [26]. TRRAP is upregulated in ovarian cancer cells and is shown to regulate the NANOG expression that maintains the stemness-like characteristics of cancer stem cells, yet in a less studied mechanism [27]. TRRAP-Tip60 co-activates the expression of the mitotic gene Top2A and promotes hepatocellular carcinoma cell proliferation [30].

Studies using mouse models have demonstrated that TRRAP is important for the transcription activation of specific genes. In mouse embryonic fibroblasts (MEFs), TRRAP recruits HAT onto the Mad1 and Mad2 promoters and thus promotes their transcription [25]. In TRRAP-deleted murine embryonic stem cells (ESCs), a low level of active chromatin markers (AcH4, H3K4me2) and a decreased transcription of stemness genes *Nanog*, *Oct4,* and *Sox2,* were detected [28]. A study using TRRAP knockout in mouse neuro-stem cells revealed that TRRAP-deletion abolished the binding of E2F1, HAT, and AcH3 onto the promoter of the cell cycle regulators Cdc25A, Mad2, CycA2, and Top2A [22]. Recently, we discovered that TRRAP mediates the binding of Sp1, a key TF, to chromatin, which promotes the hyperacetylation of the promoter region and thus induces the transcription of Sp1 target genes [29]. All of these studies demonstrate an essential role of TRRAP in the recruitment of HAT enzymes and TFs onto the target promoter to modulate proper transcription.

### 2.3. The Biological Function of TRRAP in Different Cellular and Animal Models

Studies from different model systems give a broad insight into the function of TRRAP in transcription activation and its involvement in various cellular processes. TRRAP is essential for cell viability in mammals. TRRAP null mouse blastocysts could not proliferate due to mitotic checkpoint catastrophe, resulting in a peri-implantation lethality of mouse embryos [31]. In addition, TRRAP knockout MEFs showed chromosome missegregation, mitotic exit failure, and compromised a mitotic checkpoint. TRRAP-deletion abolished the chromatin-binding of Tip60 and PCAF at the promoters of the *Mad1* and *Mad2* genes [25]. The deletion of TRRAP in ESCs resulted in an unscheduled differentiation of ESCs, likely through TRRAP’s role in HAT-mediated chromatin remodeling and the expression of the stemness marker genes, *Nanog*, *Oct4*, and *Sox2* [28]. In addition, TRRAP- deletion-induced apoptosis of the hematopoietic stem cells (HSCs) and the downregulation of c-Myc, a well-known interactor of TRRAP [23], scoring the importance of TRRAP in the homeostasis of the hematopoietic system. Clinical studies identified individuals carrying missense variants of TRRAP, who show developmental delay and neuropathological symptoms, including a variable degree of intellectual disability, autism, ASD, and epilepsy. Some of the individuals also exhibit a malformation of the cerebellum, heart, kidney, and urogenital tracks [18,32]. These case reports highlight the importance of TRRAP in organ development and neuronal functionality.

It is known that a variety of HAT or HDAC mutations lead to neurological dysfunctions and brain developmental defects in mouse models [33,34]. Using mouse models, we showed that TRRAP-HAT-mediated histone acetylation plays a vital role in brain development by regulating the expression of genes related to cell cycle progression and neural stem cell differentiation [22]. TRRAP is required for proper differentiation of neuroprogenitors during neocortical neurogenesis because its deletion lengthened the cell cycle of apical neuroprogenitor cells, rendering to a premature differentiation of neural progenitors [22]. Despite having no effects on the E2F1 protein level, TRRAP-deletion decreased the chromatin binding of E2F1 and HAT and repressed the active chromatin mark AcH3, on the promoter of cell cycle regulators, thus reducing the expression of these factors [22].

Surprisingly, when TRRAP was specifically deleted in post-mitotic Purkinje cells (PC) in a mouse model, these mutant neuronal cells were viable and contributed normally to cerebellogenesis, which nevertheless caused a progressive neurodegeneration in aged animals [29]. The integrated transcriptomic, proteomic, and epigenomic analyses identified that Sp1 is a novel TF under the control of TRRAP. We found that the TRRAP-HAT-Sp1 transactivation activity regulates the expression of genes involved in microtubule dynamics, specifically Stmn3 and Stmn4. TRRAP-deletion results in a hypoacetylation of, and simultaneously an insufficient Sp1 binding on, Stmn3 and Stmn4 promoter proximity [29]. These findings thus identify an important role of the TRRAP-HAT-Sp1 axis in the protection against neurodegeneration via the regulation of microtubule dynamics. However, whether the acetylation of the Sp1 protein per se by TRRAP-mediated-HATs directly changes Sp1 activity on the target promoter, deserves future investigations.

## 3. Sp1 Is a Ubiquitous TF

### 3.1. Overview

Sp1 is a TF that is expressed ubiquitously in all mammalian cell types [35]. It was named after its purification method, the sephacryl and phosphocellulose columns, but later renamed after specificity protein 1 [36,37]. Initially, Sp1 was considered to regulate housekeeping genes, yet later studies revealed the tissue-specific function of the Sp1-mediated transcription [38]. Perhaps, the most studied function of Sp1 is in tumorigenesis [39]. The overexpression of Sp1 emerges in various cancer types, e.g., human glioma, breast cancer, gastric cancer, pancreatic ductal adenocarcinoma, and thyroid tumors. Many Sp1 targets are the hallmarks of cancer progression, including EGF on sustained proliferation/immortality [40], Bcl-2 on apoptosis [41], TSP-1 on angiogenesis [42], MMP9 on metastasis [43], and BRCA1 on DNA damage/stress response [44].

### 3.2. Sp1 Structure

**Figure 2:**
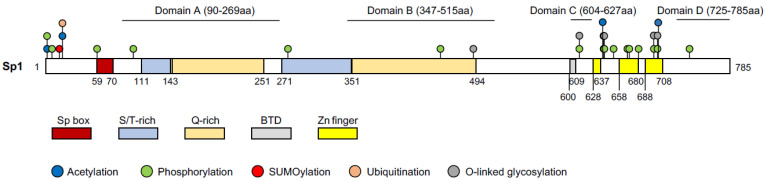
Protein structure of Sp1 with indicated domains and post-translational modifications. Domains A, B, C, and D indicate transactivation domains. All of the other domains are indicated in boxes with different colors. Putative post-translational modifications are labelled in color-coded circles. The numbers under the protein indicate the position of the amino acid at the border of the domains. S/T-rich: serine/threonine-rich domain; Q-Rich: glutamine-rich domain; BTD: Buttonhead domain; Zn finger: Cys2His2-type zinc finger domain. UniProt ID: P08047 and references [46,47,48].

Sp1 is a 785aa protein with a molecular weight of 105 kD and its structure has been well mapped [35] (Figure 2). Sp1 contains three domains: the Sp box on the N-terminus, the Buttonhead domain (BTD), and the Zinc finger domain on the C-terminus [35]. The Zinc finger domain (the DNA binding domain), consisting of three adjacent Cys2His2-type zinc finger motifs, recognizes the GC boxes (GGGGCGGGG) and the GT/CACC boxes (GGTGTGGGG) of the DNA sequence. The BTD domain is suggested to express the Sp1 activity [35]. However, a recent study showed that the absence of this domain in *Drosophila* did not affect the expression of the Sp1 targets nor fly development [45]. The Sp box contains an endoproteolytic cleavage site, which is implied in protein degradation. Sp1 has four transactivation domains; domain A and B are serine/threonine- and glutamine-rich, which are responsible for most of the transcriptional activity of Sp1. The highly charged domain C promotes the transactivation and the DNA binding of Sp1, whereas domain D supports Sp1 multimerization [35].

### 3.3. Transcription Initiation and Transactivation by Sp1

Sp1 induces the initiation of transcription through the recruitment of the basal transcription machinery to the chromatin. It specifically interacts with general human TBP and TBP-associated factor II 130 (hTAFII130) in the general transcription factor IID (TFIID) complex, which then initiates the formation of the pre-initiation complex and thus, the transcription initiation [49,50]. Sp1 expresses its activity in a synergistic way with other TFs, including E2F1, AP2, Oct-1, and Sp1 itself [51,52,53,54]. Sp1 interacts with other activators and forms the multimers through the glutamine-rich transactivation domains A and B, as well as D [55]. Even an additional binding site outside of the promoter, but in the enhancer regions, can promote the transcription activity of target genes [46]. Another evidence of the functional synergy of Sp1 at the regulatory element is that the assembly of multiple tetramers of Sp1 increases its activity [46,56].

### 3.4. Regulation of Sp1 Activity

Sp1 has been shown to interact with epigenetic modifiers to induce or repress the transcription of target genes. Sp1 recruits HAT to stimulate Sp1 transactivation. p300, as a HAT enzyme, is an interaction partner of Sp1, which facilitates the binding of Sp1 to chromatin, to initiate the transcription [57,58]. Another study focused on the effect of NGF on neuronal differentiation, unraveled the cooperation between Sp1 and p300. p300 co-activates Sp1, leading to the transcription of p21 and promoting neuronal differentiation [59]. Sp1 also harbors an inhibitory function in transcription. Sp1 recruits the repressor HDAC1 to the GM2-synthase promoter as a part of the repressor complex and thus inhibits the expression of GM2-synthase [60]. However, Sp1-mediated transcription can be complex in a way that the activating or repressing effect of Sp1 on gene regulation could be switched in a temporal–spatial manner, depending on the partners or interactors at the promoter of the target genes. For example, Sp1 regulates the transcription of 12(S)-lipoxygenase, which participates in the epidermal and epithelial inflammation, through the recruitment of HDAC1 and p300 to the promoter [61]. Upon treatment by phorbol 12-myristate 13-acetate (PMA), Sp1 is constitutively acetylated by yet unknown HATs and recruits HDAC1 to the chromatin, to deacetylate Sp1 and subsequently dissociates HDAC1 from Sp1. The Sp1 deacetylation would allow the interaction of p300 with Sp1 and the recruitment of p300 to the target promoter which then catalyzes histone acetylation and leads to target expression [61]. This study has conceptualized that the recruitment of a post-translational modifier to TFs at certain chromatin regions dictates the transcriptional activity of TFs.

### 3.5. Post-Translational Modification (PTM) on Sp1

The activity of Sp1 can be affected by various PTMs: phosphorylation [62], acetylation [57], glycosylation [63], ubiquitination [64], SUMOylation [65], and poly(ADP-ribosyl)ation [66]. The temporal–spatial interaction mode between these PTMs complexes the regulation of Sp1 activity. Depending on the kinase, phosphorylation can either increase or decrease the transcriptional activity of Sp1. Under the FGF2 treatment in mammalian cells, for instance, ERK1/2 phosphorylate Sp1 on Thr453 and Thr739, thus repressing the expression of its target, PDGFR*α* [67]. p38 kinase phosphorylates Sp1 and induces the filamin-A expression in connective tissues [68]. Compared to phosphorylation, very few HATs are known to conduct acetylation on Sp1 and the results are often inconsistent. It has been shown that Sp1 acetylation, catalyzed by p300, does not affect Sp1 binding on DNA [57]. In contrary to this, another study showed that p300 acetylates Sp1 on K703, which reduces the interaction between Sp1 and p300 and represses its transcriptional activity [61]. To date, not many acetylation residues on Sp1 have been identified, other than K703 [61], K639, K624, K685, K693 [48], and K19 [47], with K703 being the most studied. However, which residues of Sp1 are acetylated by other HATs and what the biological functions of the respective acetylation sites are, remain unknown. SUMOylation and acetylation can affect protein activity in an antagonistic manner. Moreover, SUMOylation and ubiquitination both affect Sp1 stability [69]. SUMOylation of Sp1 at K16 increases the ubiquitination and degradation of Sp1. However, phosphorylation of Sp1 inhibits the SUMOylation on K16 and thereby stabilizes Sp1 [69]. Generally, ubiquitination competes against acetylation on the common lysine residue and determines the stability. A study showed that Sp1 can be acetylated and ubiquitinated on K19 [47]; although, whether the Sp1 acetylation on K16 and/or K19 can affect Sp1 protein stability or its activity, was not reported.

### 3.6. Novel Functions of Sp1 in the Nervous System and Diseases

As a master transcription factor, Sp1 has a plethora of downstream target genes, yet, the upstream regulatory mechanism of Sp1 remained unknown until recently in a study showing that TRRAP-HAT is upstream of Sp1, to regulate the Sp1-mediated transcription [29]. A well-documented function of Sp1 is in cell proliferation of cancer [39]. Moreover, Sp1 has been linked with the neuropathologies of the nervous system, albeit some controversies. The GWAS analysis implicates that Sp1 mediates transcriptional activity changes in patients suffering from the neurodegenerative disorders, Alzheimer’s disease (AD) and Parkinson’s disease (PD) [70]. Sp1 is upregulated in AD patients and AD mouse models; however, the chemical inhibitor of Sp1 even led to severe memory deficits in AD transgenic mice [71]. The occurrence of Huntington’s disease (HD) results from the mutation of the huntingtin protein, which contains an aberrant poly-glutamine tract. The mutant huntingtin was shown to disrupt the interaction between Sp1 and the co-activator TAFII130, thereby inhibiting the transcription of the dopamine D2 receptor, a hallmark of HD [72]. Further studies in the HD mouse model demonstrated that mutant huntingtin inhibits ZnT3 transcription through the interaction with Sp1, causing a loss of the synaptic vesicular zinc molecule in the neurons of the CA1, CA2, and CA3 region of the hippocampus, cortex, and striatum [73]. Synaptic vesicular zinc modulates synaptic transmission and the maintenance of cognitive capacity in the prevention of the HD pathology [73]. However, it is also reported that a downregulation of Sp1 is protective in HD development [74]. These controversial findings, although confirmed a role of Sp1 in neuropathies, can be explained by the complex mechanism of Sp1 activity regulation, such as transcriptional regulation, epigenetic, and posttranslational modifications [38]. It is also possible that Sp1 behaves differently in the manifestation of the disease processes in a very heterogenous genetic background in human studies.

Despite the controversy, Sp1 has been shown, in neural model studies, to bind to the promoters of neural genes, e.g., Slit2 [75], P2X7 [76], and Reelin [77]. Our transcriptome studies identified various neurological processes that are regulated by Sp1 downstream targets [29]. Many of these processes are linked with microtubule dynamics, which are closely related to neuronal homeostasis and neurodegenerative processes [78]. Specifically, Sp1 binds to the promoter of genes encoding the microtubule destabilizing proteins Stmn3 and Stmn4, and in the absence of TRRAP, Sp1 activity in the expression of Stmn3/4 was greatly compromised [29]. These findings disclose the involvement of Sp1 in neurodegenerative processes and also implicate that the miss-regulation of Sp1 activity by upstream modulators can be an etiological mechanism of neuropathological phenotypes.

## 4. Outlook

TRRAP has been shown to control gene expression in different cell types, via its role in recruiting and activating the HAT-mediated TF activity. In different cell types, TRRAP regulates different TFs that control cell cycle progression and cell differentiation. However, whether all of these involve Sp1, is unclear. It has been shown that Sp1 is upstream of c-Myc, a partner of TRRAP. E2F1 is another TRRAP interactor and has been shown to interact with Sp1 to grant its transcriptional activity [79,80]. Thus, the TRRAP-Sp1 axis might be the upstream regulator on all of the observed phenotypes in different cell lineages.

TRRAP is required for strengthening the binding of E2F1 and HATs on chromatin. This is analogous to the situation of p300, which is required for the Sp1 binding on the chromatin. However, TRRAP can act as a scaffold to accommodate TFs and HATs onto the chromatin but may also mediate the acetylation directly on TFs which may affect the Sp1 binding on chromatin, or simply alter the TF activity. This hypothesis may be supported by observations showing that lysine acetylation affects the stability and activity of TFs [81]. In addition, the TRRAP-HAT-mediated acetylation of Sp1 could compete against other PTMs, for example, ubiquitination or SUMOylation, and thus, affects Sp1 stability. Despite some of the molecular hits, the significance and function of these PTMs of Sp1 have not been well studied. Finally, targeting the TRRAP-HAT-Sp1 axis would be a potential strategy for pharmaceutical intervention, aiming at the prevention and treatment of neurodegenerative diseases.

## Figures and Tables

**Table 1:** TRRAP target genes and their respective involvement in cellular processes.

TRRAP Target Genes	Cell Type	Cellular Process and Reference
ABCA1, ABCG1, SCD, HBP	Hepatic cell lines	Lipid metabolism [16]
APOC3	Hepatocytes	Triglyceride metabolism [21]
Cdc25A, CycA2, TopA2	Neural progenitors	Stem cell differentiation [22]
CyclinD2, ID2, MCM7	Hematopoietic stem cells	Maintenance of the hematopoietic stem cell pool [23]
H2B, H4	HEK293T cells	G1/S-phase transition [24]
Mad1, Mad2	Embryonic fibroblasts	Cell cycle progression [25]
MCIDAS, CCNO, MYB	Airway epithelial cells	Multiciliated cell formation [26]
NANOG	Ovarian cancer cells	Tumorigenic potential of ovarian cancer stem cells [27]
Nanog, Oct4, Sox2	Embryonic stem cells	Maintenance of cell stemness [28]
STMN3, STMN4	Postmitotic neurons	Microtubule dynamics [29]
TOP2A	Hepatocellular carcinoma cells	Proliferation of tumor cells [27]
